# Evolutionary hierarchies of conserved blocks in 5'-noncoding sequences of dicot *rbcS *genes

**DOI:** 10.1186/1471-2148-7-51

**Published:** 2007-04-02

**Authors:** Katie E Weeks, Nadia A Chuzhanova, Iain S Donnison, Ian M Scott

**Affiliations:** 1 Cardiff School of Computer Science, Cardiff University, Queen's Buildings, 5 The Parade, Roath, Cardiff, CF24 3AA, UK; 2 Department of Biological Sciences, University of Central Lancashire, Preston, PR1 2HE, UK; 3 Institute of Grassland and Environmental Research, Aberystwyth, Ceredigion, SY23 3EB, UK; 4 Institute of Biological Sciences, University of Wales, Aberystwyth, Ceredigion, SY23 3DA, UK

## Abstract

**Background:**

Evolutionary processes in gene regulatory regions are major determinants of organismal evolution, but exceptionally challenging to study. We explored the possibilities of evolutionary analysis of phylogenetic footprints in 5'-noncoding sequences (NCS) from 27 ribulose-1,5-bisphosphate carboxylase small subunit (*rbcS*) genes, from three dicot families (Brassicaceae, Fabaceae and Solanaceae).

**Results:**

Sequences of up to 400 bp encompassing proximal promoter and 5'-untranslated regions were analyzed. We conducted phylogenetic footprinting by several alternative methods: generalized Lempel-Ziv complexity (*C*_*LZ*_), multiple alignments with DIALIGN and ALIGN-M, and the MOTIF SAMPLER Gibbs sampling algorithm. These tools collectively defined 36 conserved blocks of mean length 12.8 bp. On average, 12.5 blocks were found in each 5'-NCS. The blocks occurred in arrays whose relative order was absolutely conserved, confirming the existence of 'conserved modular arrays' in promoters. Identities of half of the blocks confirmed past *rbcS *research, including versions of the I-box, G-box, and GT-1 sites such as Box II. Over 90% of blocks overlapped DNase-protected regions in tomato 5'-NCS. Regions characterized by low *C*_*LZ *_in sliding-window analyses were also frequently associated with DNase-protection. Blocks could be assigned to evolutionary hierarchies based on taxonomic distribution and estimated age. Lineage divergence dates implied that 13 blocks found in all three plant families were of Cretaceous antiquity, while other family-specific blocks were much younger. Blocks were also dated by formation of multigene families, using genome and coding sequence information. Dendrograms of evolutionary relations of the 5'-NCS were produced by several methods, including: cluster analysis using pairwise *C*_*LZ *_values; evolutionary trees of DIALIGN sequence alignments; and cladistic analysis of conserved blocks.

**Conclusion:**

Dicot 5'-NCS contain conserved modular arrays of recurrent sequence blocks, which are coincident with functional elements. These blocks are amenable to evolutionary interpretation as hierarchies in which ancient, taxonomically widespread blocks can be distinguished from more recent, taxon-specific ones.

## Background

Promoter sequences have been described as a vast and largely uncharted territory for evolutionary biologists [1]. One impediment to exploration is the difficulty of motif prediction in noncoding sequences (NCS): motif-discovery tools achieved detection rates of only 22–35% for transcription factor (TF) binding sites in recent benchmark studies [2,3]. Although it has long been recognized in principle [4] that evidence for motifs can be enhanced by comparing sequences of common ancestry, 'phylogenetic footprinting' of higher eukaryotes is still in a development and evaluation phase [5-8]. There are also perceived challenges in the use of sequence alignment for phylogenetic analysis of NCS [9], as complex mutational processes (slipped-strand mispairing, stem-loop secondary structure excision/repair, minute inversions, intramolecular recombination) are prevalent. In practice, however, Bremer et al. [10] found chloroplast NCS to be of similar utility to coding sequences in phylogenetic tree construction for asterids. This result confirmed that plant NCS contain evolutionary signal, which might be hypothesized to reside in the conserved motifs sought in phylogenetic footprinting. The present study sought to explore the extent to which phylogenetic footprints in plant 5'-NCS could be subjected to evolutionary analysis and interpretation. For this objective, we needed to conduct sufficiently comprehensive phylogenetic footprinting for meaningful evolutionary analysis of conserved sequence blocks.

We employed a greater taxonomic range than other phylogenetic footprinting studies of plant NCS, which have been confined to single families [6,8,11,12] or to a couple of species [13]. Much of the interest in promoter evolution lies in comparisons of paralogous genes (i.e. genes that diverged after a duplication event). In consequence, it must be noted, our dataset included several multigene families, and therefore was not optimized to investigate taxonomic phylogenies in the manner of Bremer et al. [10].

Recognizing limitations in individual motif discovery tools [2,3,7], we sought to maximize detection of conservation by combining distinct methodologies. Analysis of generalized Lempel-Ziv complexity (*C*_*LZ*_), played several roles in our study. *C*_*LZ *_measures the complexity of a text as the minimal number of steps in a defined procedure of its synthesis with the parsing rule: the next phrase is the longest seen previously. Many text compression algorithms are based on Lempel-Ziv parsing [14]. Computation of *C*_*LZ *_thus involves a decomposition of the text into repeated blocks, and an application to the discovery of structural regularities in genetic 'texts' was realized by Gusev et al. [15]. This method has identified arrays of conserved sequence blocks in NCS of vertebrates from fish to humans [16,17]. *C*_*LZ *_analysis has also been used to study human mutagenic mechanisms [18,19] and genomic architecture [20].

Our second tool was MOTIF SAMPLER, in which the probability of finding a particular motif is estimated using Gibbs sampling and modelling of the background sequence with a Markov model [21].

We complemented these tools with sequence alignment, including the DIALIGN and ALIGN-M algorithms designed for highly divergent sequences with only localized similarities, as seen in 5'-NCS. DIALIGN is based on a segment-to-segment comparison [22,23], while ALIGN-M uses a non-progressive local approach to guide alignments [24].

We focused on 5'-NCS of ribulose-1,5-bisphosphate carboxylase small subunit (*rbcS*) genes because of the exceptional corpus of knowledge against which analytical outcomes could be benchmarked. As the earliest nuclear protein-coding sequences in plants to be cloned, *rbcS *genes became paradigms for functional studies of plant promoters [25], and several classes of *cis*-elements were originally defined in *rbcS *promoters. Thus, the prototype of trihelix TFs was the nuclear protein GT-1, which binds to the 14-bp Box II and related motifs in light-responsive regions of the pea *rbcS-3A *promoter [26]. Box II versions featured in the earliest *rbcS *promoter alignments [25,27], and occur in other light-responsive genes [28], where they may be targets of calcium/calmodulin phototransduction [29].

Two further *cis*-elements discovered in *rbcS *promoters, the G-box and I-box, are common features in light-responsive promoters [28,30], and have been functionally characterized as dual components of a minimal light-responsive unit [31]. G-box binding factors (GBFs), identified using tomato *rbcS-3A *upstream sequences [32,33], are basic leucine zipper TFs interacting with the G-box core, CACGTG [34]. Dicot *rbcS *G-boxes interact with the HY5 GBF, which mediates phytochrome and cryptochrome signals in concert with COP and DET regulators [31,35].

The I-box, core motif GATAAGR, was also defined in *rbcS *promoters [27,33,36]. Its reverse, YCTTATC, was highlighted in *rbcS *and other light-regulated promoters by early motif searches [37,38]. Binding factors for the I-box are still being clarified. Functional interactions occurred in yeast between I-box sequences and recombinant zinc-finger GATA TFs from *Arabidopsis *[39]. I-box binding nuclear proteins reported in several species [40,41] may therefore include GATA TFs, though the first cloned I-box binding protein was a tomato Myb-like TF [42]. While the above *rbcS cis-*elements are the most studied, there is evidence for numerous further elements and DNA-protein interactions in *rbcS *promoters [30,32,43-48].

There is a particularly extensive history of characterization of *rbcS *promoters from pea, *Petunia *and tomato [25,49]. We analyzed these along with other studied *rbcS *5'-NCS such as those of *Arabidopsis *[50] to provide a gradation of taxonomic relations and evolutionary distances. Conserved features shared by the plant families analyzed would have persisted since the Cretaceous, to which can be dated the divergence of eurosids I (represented by the Fabaceae) from eurosids II (Brassicaeae), and both from asterids (Solanaceae) [51].

## Results

### Phylogenetic footprinting

5'-NCS of up to 400 bp including proximal promoter and 5'-untranslated regions (5'-UTRs) were analyzed for 27 dicot *rbcS *genes. The rosid complement comprised all four *Arabidopsis *genes (three from a tandem locus), plus genes from *Brassica *and the legumes *Phaseolus*, *Medicago *and *Pisum *(pea). The *Lycopersicon *(tomato), *Solanum *(potato), *Petunia *and *Nicotiana *genes included representatives of all three solanaceous *rbcS *loci, which are distinguished by features including an extra (third) intron in 'locus 2' genes, and tandem duplicates at 'locus 3' [25]. Phylogenetic footprinting analyses were performed on the entire dataset, and separately on various subgroups, e.g. rosid, brassica, legume, or solanaceous genes, or genes of each solanaceous locus. Three methodologies were employed:

(1) *C*_*LZ *_analysis was used as proposed by Gusev et al. [15] to search for recurrent sequence blocks in the *rbcS *5'-NCS. The *C*_*LZ *_measure is based on representation of a sequence by fragments that have been encountered before (in the same or other sequences). Let *S *= *s*_1 _... *s*_*L *_be a nucleotide sequence of length *L*. Denote by *S *[*i*:*j*] the substring of *S *that starts at position *i *and ends at position *j*. A Lempel-Ziv decomposition of *S *is a partition of *S *into *m *consecutive fragments, *S *= *S *[1:*i*_1_] *S *[*i*_1_+1:*i*_2_]...*S *[*i*_*m*-1_:*L*], such that the *k*-th component *S *[*i*_k-1_+1:*i*_*k*_] is the longest fragment downstream of position *i*_*k*-1 _for which an exact repeat has been encountered somewhere upstream of position *i*_*k*-1_+1. The number of fragments in the decomposition, *C*_*LZ *_(*S*) = *m*, is called the complexity of *S *with respect to direct repeats. For example, if *S *= TCGATCGAGAT, then the decomposition of *S *with respect to direct repeats is T-C-G-A-TCGA-GAT. Fragments 1, 2, 3 and 4 in this decomposition are of length one since respective nucleotides T, C, G and A occur for the first time. Exact copies of fragments 5 and 6 occur in positions 1 and 3 respectively. The *C*_*LZ *_of the sequence with respect to direct repeats equals 6. To find fragments repeated in different *rbcS *5'-NCS, we concatenated multiple sequences for *C*_*LZ *_analysis.

(2) Overrepresented motifs were sought with MOTIF SAMPLER, using a range of program options for prior probabilities, lengths, numbers and overlaps of motifs. MOTIF SAMPLER can also vary the background Markov model order (i.e. dependency on a given number of preceding sequence positions). Thijs et al. [52] found higher order models improved robustness of motif recovery in *Arabidopsis *data. We found that the optimal Markov model order differed for different motifs: in 40 repeat runs, optimal model orders were zero for detection of blocks 06, 22 and 29, first for 10, 25 and 30, second for 23 and 28, and third for 08 and 20. (Blocks are defined in Table 1.)

**Table 1:** Conserved Blocks in *rbcS *5'-NCS

Block	Definition^a^	Associated motifs^b^
01	GCGTCTGATTT	(?)ARR1 site
02	AAGGAGCCAAAAGC	(?)Dof site
03	AACCGATCAAGTGGAGA	(?)MYC site
04	AAAAATGAAAAACTTGTC	(?)GT-1 site
05	AACCATACACA	(?)MYB site
06*	ATCACACATT	*rbcS *Box III* [32]
07	ATATCCTCTTCCTACCCCCAT	(?)PHR1 site; (?)MYB site
08*	GATGAGATAAGA	*rbcS *I-box [31]; *rbcS*-CMA5 [28]
09*	TTTGAGATAAGGA	*rbcS *I-box [31]; Manzara 5 [27]
10*	ACACGTGGCA	*rbcS *G-box [31]; *rbcS*-CMA4/5 [28]
11*	TCCTATTGGTGGCT	*rbcS*-CMA4 [28]; Manzara 4/8 [27]
12*	GATAAGGCT	*rbcS *I-box [31]; *rbcS*-CMA4 [28]
13	TCAACACCTTTCCTT	(?)RAV1-A site; (?)Dof site
14	GGCACTTAGCTCCAATT	(?)CCAAT-box
15	TTTCCAACC	(?)MYB site
16*	AGGGGTTAAA	Manzara 6 [27]; (?)GT1-core [32]
17*	ATCTTGTGTGGTTAAT	*rbcS *Box II [32]; *rbcS*-CMA3 [28]; Manzara 8 [27]
18	AACGACGTTATCATGAAT	(?)ACGT element; (-)I-box [27]
19*	GCAAAGTTT	*rbcS *3AF-5 site [43]; *rbcS*-CMA3 [28]
20*	TGTAATGTCA	Manzara 9 [49]; (?)(-)W-box
21*	ATCATTTTCAC	*rbcS *Box III [32]; *rbcS*-CMA3 [28]
22*	CCACATAA	*rbcS*-CMA2 [28]; Manzara 10 [49]
23*	TCCAATGGTTA	*rbcS*-CMA2 [28]; Manzara 12–13 [49]; CAAT-box
24	ACCCTTTGATCATTA	(?)(-)Dof site
25*	TCTAAGATGAGGTTTGCT	*rbcS*-CMA2 [28]; Manzara 15 [49]
26	TACCACAATTT	(?)CAAT-box
27	ACCATAATATTGGAA	*rbcS*-CMA1 [28]; (?)(-)CCAAT-box
28*	TTGTGTCCGTTAGATG	Manzara 16 [49]; (?)MYB site
29*	CCTTATCAT	*rbcS*-CMA1 [28]; LRE [49]
30*	TATATAAA	*rbcS*-CMA1 [28]; TATA-box [49]
31	GAGGGGGA	(?)WT-1 site
32	ATGACAAAACCA	(?)W-box; (?)MYB site
33*	AAGCTTTGCAA	*rbcS *Box V [90]; (?)(-)Dof site
34	GCAATAACCCTCTT	(?)CAAT-box
35	AAGAAGAAGA	-
36	TTTTCAGCA	-

^a^Typical instances (full list in Additional File 1). ^b^(?)PLACE database match; (-)reverse-strand motif. *Noted previously in *rbcS *genes: references in square brackets.

(3) Sites of local congruence were sought in multiple sequence alignments produced by CLUSTALW, ALIGN-M and DIALIGN, with various gap penalty options for the first two. Collation of methodologies by mapping output from *C*_*LZ *_and Gibbs sampling analyses onto alignments yielded useful synergies. In particular, the alignments revealed arrays of blocks that occurred in several sequences in the same order, which increased confidence in less conserved block versions that occurred in the appropriate position relative to other blocks.

Our initial *C*_*LZ *_procedure specified blocks ≥ 8 bp with up to two mismatches, which identified 218 instances of 34 conserved blocks (on average 90% identical with their definitions in Table 1). Relaxation of the mismatch criterion for DIALIGN-aligned versions of these 34 *C*_*LZ*_-defined blocks exposed an additional 109 instances (of average 76% identity with definitions).

Conversely, mapping blocks from other tools clarified often complex alignments. When the full dataset was aligned by DIALIGN, 67% of aligned blocks split into an average 3.5 fragments, and 86% of blocks were co-aligned on average with 1.7 others. Nonetheless, with support from *C*_*LZ *_and MOTIF SAMPLER, 323 instances of 35 blocks were identified within alignments. MOTIF SAMPLER used independently found 291 instances of 35 blocks.

**Figure 1:**
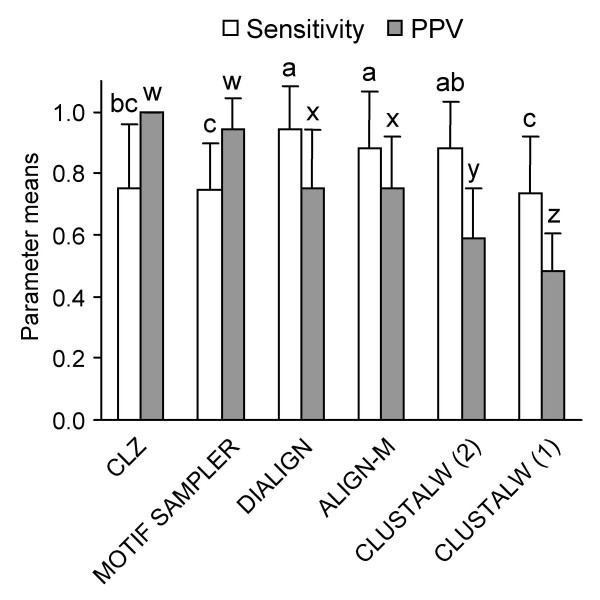
**Comparison of phylogenetic footprinting tools in predicting the 12 most frequent blocks in the 27 dicot *rbcS *5'-NCS**. See text for Sensitivity (equation 1) and PPV (equation 2) performance parameters. MOTIF SAMPLER was run 8 times each for background model orders 0–3, and with the prior probability of motif (*p*) at 0.3, the empirical value from the analytical consensus. Other option settings were *s *0, *M *1, *n *3, *w *11, *x *0, *r *5. Gap penalties for CLUSTALW (1) were: opening 15.0, extension 6.66; and for ALIGN-M and CLUSTALW (2): opening 8.0, extension 0.5. Mean performance parameters shown with standard deviation bars. Values sharing alphabet labels were not significantly different (Mann-Whitney *U *test, *P *> 0.05).

The complementarity of our different phylogenetic footprinting methods was demonstrated by the benchmarking exercise in Figure 1. In this exercise, each tool independently analyzed the full set of 27 5'-NCS, to test performance (versus the methodological consensus) in scoring each instance of the 12 most frequent blocks. Performance parameters, following Tompa et al. [3], were:

Sensitivity = *nTP*/(*nTP*+*nFN*)

Positive Predictive Value (PPV) = *nTP*/(*nTP*+*nFP*)

where *nTP *= number of 'true' positives (identified blocks found also by other tools), *nFN *= 'false' negatives (blocks not found though supported by other tools), and *nFP *= 'false' positives (blocks found but not supported by other tools). (Since every block instance had not been verified as a *cis*-element, the 'true' and 'false' concepts in these equations reflected sequence analysis performance rather than functionality prediction.)

*C*_*LZ *_analysis and MOTIF SAMPLER showed greater PPV in block prediction, but weaker sensitivities, than the best alignment methods (Figure 1). MOTIF SAMPLER's sensitivity for individual blocks correlated (*r *= 0.85, *P *< 0.001) with its log-likelihood statistic [21] that is optimized during Gibbs sampling. Among the alignment tools, DIALIGN and ALIGN-M, designed for highly divergent sequences with localized alignments, outperformed the CLUSTALW global alignment algorithm (Figure 1). The performance of CLUSTALW was significantly improved by reducing the gap penalties, though the PPV of DIALIGN and ALIGN-M remained superior (Figure 1).

MOTIF SAMPLER outputs statistical data, which helped estimate the significance of our phylogenetic footprinting results. Ten dummy datasets with different randomizations of every sequence were analyzed by MOTIF SAMPLER using background model orders 0–3. Randomization caused MOTIF SAMPLER to find on average 6.5-fold fewer pseudo-motif instances. Log-likelihood scores [21] for pseudo-motifs in the 10 dummy datasets were much lower (mean = 49.6, standard deviation = 17.3) than those of the original sequence motifs (mean = 188.7, standard deviation = 60.2), which differed from random at significance levels of *P *< 0.0001 (Kruskal-Wallis tests).

In summation, phylogenetic footprinting defined 36 conserved blocks, representing contiguous nucleotide sequences occurring in two or more *rbcS *5'-NCS, and being of sufficient length, sequence fidelity and positional similarity to make their common evolutionary origin probable. A total of 338 instances of these blocks were identified in the dataset. A large majority (275 instances of 33 blocks) were supported by all three methodologies. Of these 33 blocks, another 37 instances were supported only by *C*_*LZ *_and alignments, and 5 more only by *C*_*LZ *_and MOTIF SAMPLER. Two other blocks (11 instances) were defined using only MOTIF SAMPLER and alignments, and a single block (10 instances) only by *C*_*LZ *_analysis.

### Conserved blocks

**Figure 2:**
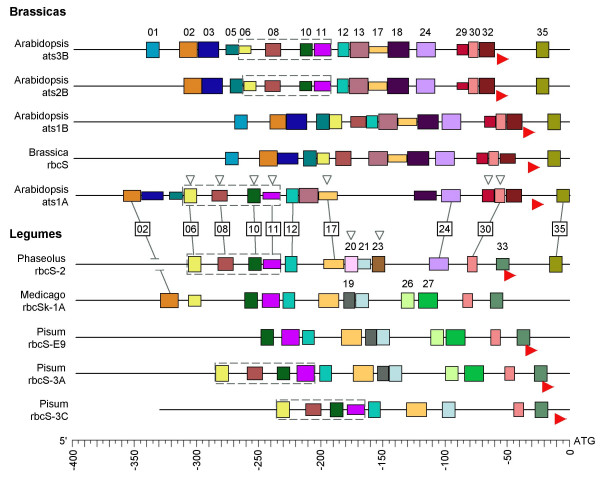
**Block structures of rosid *rbcS *5'-NCS**. Blocks individually coloured, and numbered (as Table 1) on first appearance from top. Horizontal dimension = block length (bp), vertical dimension proportional to identity with definitions in Table 1 (range: 40–100%). Complete [06-08-10-11] CMAs in dotted-line boxes. Blocks common to brassica and legume 5'-NCS joined by lines with block numbers in boxes. Blocks also found in solanaceous 5'-NCS indicated by unfilled arrowheads on *Arabidopsis ats1A *and *Phaseolus rbcS-2*. Red arrowheads show experimentally determined transcription start sites [46, 50, 86, 87].

**Figure 3:**
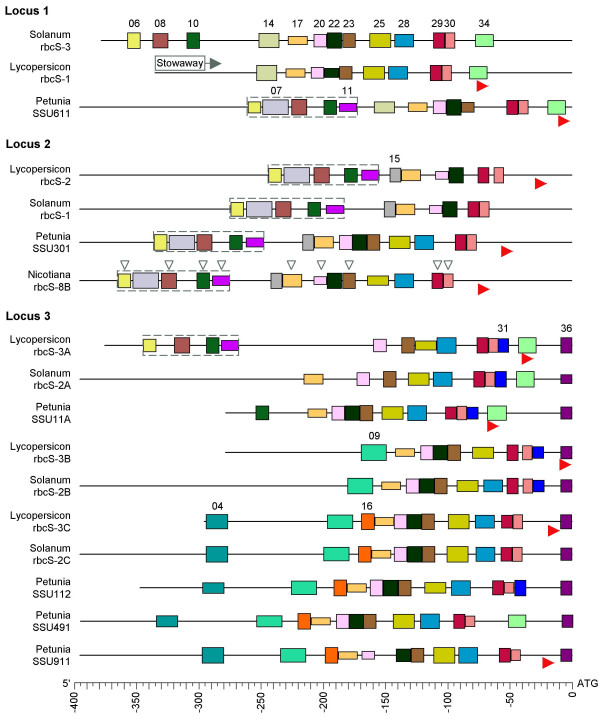
**Block structures of solanaceous rbcS 5'-NCS**. Sequences grouped as the 3 loci of Dean et al. [25]. Blocks individually coloured, and numbered (as Table 1) on first appearance from top. Horizontal dimension = block length (bp), vertical dimension proportional to identity with Table 1 definitions (range: 47–100%). Complete [06-08-10-11] CMAs in dotted-line boxes. Blocks also in rosid 5'-NCS indicated by unfilled arrowheads on *Nicotiana rbcS-8B*. Red arrowheads show experimentally determined transcription start sites [53, 54, 88, 89]. *Stowaway-Le2 *transposable element is mapped in tomato *rbcS-1 *[56].

All block instances are mapped for the rosid (brassica and legume) 5'-NCS in Figure 2, and solanaceous 5'-NCS in Figure 3. An average of 12.5 blocks were found in each gene. The blocks occurred in arrays whose relative order was absolutely conserved, so that the number-codes detailed in Table 1 consistently reflect relative block positions from 5' to 3' in all sequences. We therefore confirmed observations of Argüello-Astorga and Herrera-Estrella [28] on the existence in light-responsive plant promoters of 'conserved modular arrays' (CMAs), which they defined as 'short promoter regions, including at least two different DNA stretches larger than 6 bp (putative individual factor binding sites or phylogenetic footprints), in which nucleotidic sequence, spacing, and position relative to the transcription start site are conserved in a phylogenetic series'.

Over a third of blocks were conserved in two or more plant families, but the remainder were distinctive to single families, or, in the case of the solanaceous genes, to particular orthologous loci identified by Dean et al. [25].

Blocks are listed in Table 1 with 'definitions' as typical instances, since for variable blocks a consensus would be dominated by ambiguous IUPAC code. The degree of conservation of each instance relative to the 'definition' is indicated by the vertical block dimensions in Figures 2 and 3; the 'definitions' were chosen to maximize these dimensions and do not necessarily represent importance in functional terms. Full sequences and locations of all block instances are in Additional File 1.

The 18 blocks asterisked in Table 1 have been recognized in past *rbcS *research. Of these, the motif most represented was the I-box (blocks 08, 09, 12, 18, 29). The reverse-strand I-box (block 29) immediately upstream of the TATA-box (block 30) was found by Grob and Stüber [37], who termed it the light-responsive element (LRE).

The I-box block 08 functions in a light-responsive dual unit with the G-box block 10 [31]. The I-G boxes unit represented by blocks [08–10] was found to be common in light-responsive promoters, and termed *rbcS*-CMA5 by Argüello-Astorga and Herrera-Estrella [28]. In rosid NCS, a second I-box downstream (block 12) occurred in an I-G-I boxes array postulated as ancestral by these authors. The TG-rich block 11, between the G-box (10) and second I-box (12), formed part of *rbcS*-CMA4 of Argüello-Astorga and Herrera-Estrella [28]. Block 11 usually corresponded to Motif 4 of Manzara and Gruissem [27] (but see later on Box II).

In the present study, the largest CMA found in all three plant families comprised blocks [06-08-10-11], in dotted-line boxes in Figures 2 and 3. Block 06 is a previously overlooked motif, but we found identical versions in similar relative locations in the caryophyllid genes *Mesembryanthemum crystallinum rbcS-1 *[EMBL L10212, -241 bp] and *Spinacia oleracea rbcS-1 *[EMBL X73236, -363 bp]. In pea *rbcS-3A*, block 06 overlapped the 5' flank of the box III* inverted GT-1 site [32], and so might be a site for a factor like 3AF5, a light-regulated phosphoprotein that binds the 5' flank of the similar downstream Box III [43]. The pea *rbcS-3A *3AF5 and Box III sites themselves corresponded to legume-specific blocks 19 and 21, which with block 17 are equivalent to *rbcS*-CMA3 of Argüello-Astorga and Herrera-Estrella [28].

Block 17 coincided with the pea *rbcS-3A *Box II element, which is the prototype of GT-1 trihelix TF binding sites and a target of the calcium phototransduction pathway [26,29]. The variability of Box II-like motifs [28] was reflected in the low MOTIF SAMPLER consensus score [21] for block 17 (1.04), but this block was recognized by MOTIF SAMPLER with 85% sensitivity, and aligned in all dicot NCS by DIALIGN and ALIGN-M on its relatively conserved TGTGG sub-fragment. The Box II motifs of earlier alignments [25,27] corresponded to block 17 for most sequences, but to block 11 for tomato *rbcS-2 *and *rbcS-3A*. (Local alignments of sequences not available to the earlier authors confirm our assignments.)

The solanaceous 5'-NCS (Figure 2) yielded further previously identified motifs, whose functions generally remain uncertain. Blocks 22, 23 and 25 were components of *rbcS*-CMA2 [28] and identified by Manzara et al. [49] (Table 1). Likewise, the blocks 20 and 28 flanking *rbcS*-CMA2 were found by Manzara et al. [49] (Table 1).

On average, 10.2% of the length of those sequences with known transcription start sites was occupied by 5'-UTRs, though these were highly variable in extent (Figures 2, 3). Blocks 32–34 occurred in the proximities of transcription start sites. Only two blocks, 35 and 36, were located fully within 5'-UTRs, but each featured in multiple sequences in several species (Figures 2, 3).

Precisely half the blocks in Table 1 were newly identified in this study. These novel blocks were confined to single plant families, apart from the brassica blocks 02, 24 and 35 also found in a legume species. In most of the novel blocks, potential *cis*-elements could be speculatively identified using promoter databases (Table 1).

**Figure 4:**
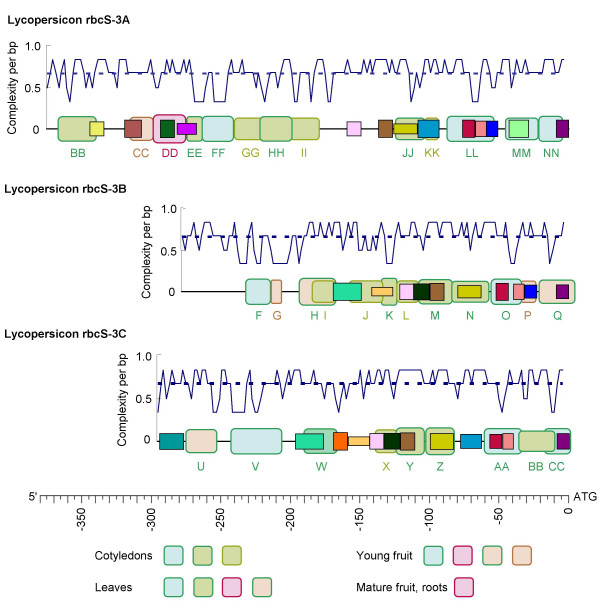
**Protein binding relative to sequence structure in rbcS 5'-NCS of tomato locus 3**. Round-cornered rectangles with alphabet labels correspond to mapped regions of DNase protection, colour-coded by organ [53, 54]. Square-cornered rectangles are conserved blocks as Figure 3. Line plots show local *C*_*LZ *_bp^-1 ^with respect to the [AT][GC] alphabet in 6-bp sliding-window profiles in 2-bp steps.

Protein-DNA interactions in tomato *rbcS *5'-NCS have been extensively mapped by Gruissem and colleagues, using DNase I footprinting of promoter fragments in nuclear extracts from different organs [49,53,54]. As shown for locus 3 (Figure 4), over 90% of our conserved blocks overlapped with DNase-protected regions in the 5'-NCS where these authors had defined DNase footprints for both DNA strands. DNase-protected regions also included blocks 31, 34, 36, which have not been defined in past studies.

On the other hand, one-third of DNase-protected regions did not overlap with well defined blocks (Figure 4). These additional DNase-protected sequences tended to be very variable between genes and dominated by particular nucleotides (e.g. AT-rich regions). The latter feature can be formally translated as low complexity, as shown by the sliding-window profiles of *C*_*LZ *_[55] with respect to the [AT] [GC] alphabet in Figure 4. The association of DNase-protection with *C*_*LZ *_troughs implied functional roles for low-complexity regions.

One characterized mechanism for the introduction of AT-richness (and thus low *C*_*LZ*_) into an *rbcS *5'-NCS sequence is the *Stowaway-Le2 *inverted repeat element in the tomato *rbcS-1 *sequence (Figure 3) [56,57]. Sliding-window *C*_*LZ *_profiles confirmed the *Stowaway-Le2 *sequence as one of the main low-complexity regions of the tomato *rbcS-1 *5'-NCS (not shown). DNase-protected regions do occur within the *Stowaway-Le2 *sequence [53,56,57].

### Evolutionary analysis

**Figure 5:**
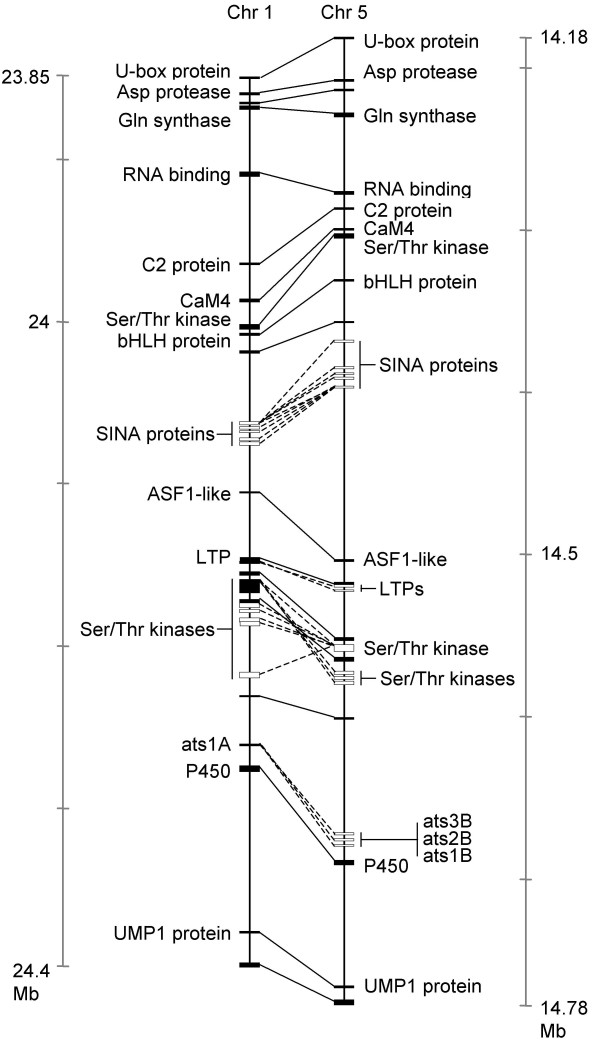
**Duplicated segment pairs on *Arabidopsis *chromosomes 1 and 5 encompassing *ats1A *and *B *family genes**. Duplicate genes linked by inter-chromosome lines, with dotted lines for tandem arrays. Gene labels are for clarity, and may refer merely to putative functions. Based on block 0105451100840, PARALOGONS IN *ARABIDOPSIS THALIANA *database [73].

The absolutely conserved relative order of blocks indicated common ancestry of all the dicot 5'-NCS studied (Figures 2, 3). This provided basic confirmation of the potential for evolutionary analysis of phylogenetic footprints, as these must share the evolutionary history of the plant taxa or gene loci with which they are associated. Minimum ages for blocks found in different species were estimated by reference to molecular clock dates for relevant taxon divergences (Table 2). For blocks common to paralogous loci, further evidence on minimum ages was available from recent studies of ancestral genome duplications (Table 2). Blanc et al. [58] produced a database of 45 duplicate chromosome segment pairs in the *Arabidopsis *genome, one of which (Figure 5) encompassed the *ats1A *and *B *genes. Comparisons of synonymous substitutions (*Ks*) in the duplicate genes indicated the relevant polyploidy event was roughly twice as ancient as the *Brassica*-*Arabidopsis *divergence [58]. Bowers et al. [59] similarly identified a duplication event, prior to the *Brassica*-*Arabidopsis *split, that generated 34 chromosome segment pairs, of which their segment α 25 encompassed the *ats1A *and *B *genes.

**Table 2:** Minimum Age Estimates for *rbcS *5'-NCS Blocks

Blocks	Occurrence	Minimum age (10^6 ^years)	Calibration events
04, 07, 09, 14, 15, 16, 22, 25, 28, 31, 34, 36	Solanaceae	18–23	Duplication of ancestral *rbcS *loci [62]; divergence of *Petunia *clade [51]
19, 26, 27	*Pisum *&*Medicago*	24	*Pisum-Medicago *divergence [91]
01	Brassicaceae	24	*Arabidopsis*-*Brassica *divergence [92]
02, 03, 05, 13, 18, 32	Brassicaceae	48	Duplication of ancestral *ats *loci [58]
21, 33	Fabaceae	54	Divergence of *Phaseolus *clade [91]
02, 12, 24, 35	Brassicaceae, Fabaceae	105	Divergence of eurosids I & II [51]
06, 08, 10, 11, 17, 20, 23, 29, 30	Brassicaceae and/or Fabaceae, Solanaceae	125	Divergence of rosids & asterids [51]

Within the duplicate segments containing the *ats1A *and *B *genes, the latter were among several examples of tandem arrays, others including LRK10L receptor-like Ser/Thr kinases [60]. Such tandem arrays, presumed due to unequal crossing over, account for up to 17% of all *Arabidopsis *genes [61], but their age range is currently uncertain [62].

**Figure 6:**
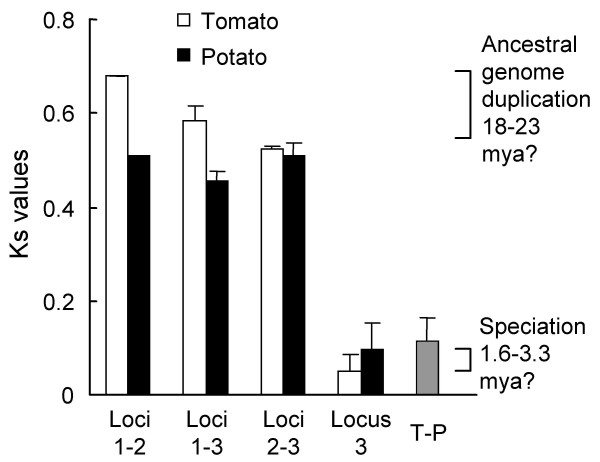
**Levels of synonymous substitutions (*Ks*) in solanaceous *rbcS *coding sequences**. Mean *Ks *(with standard deviation bars) are shown for comparisons of: all gene pairs from the 3 loci within tomato (white bars) or potato (black bars); or all paired tomato-potato (T-P) orthologues (grey bar). Brackets indicate *Ks *distribution peaks attributed by Blanc and Wolfe [62] to genome duplication or speciation events.

In the Solanaceae, a large-scale genome duplication was dated to 18–23 million years ago (mya) from *Ks *distributions of duplicate tomato and potato genes [62]. *Ks *values for inter-locus comparisons of tomato *rbcS *coding sequences were consistent with formation of the 3 loci in this event (Figure 6). This must have occurred in a common ancestor, as *Ks *values for tomato and potato *rbcS *orthologues (Figure 6) were consistent with the much more recent speciation date estimated at 1.6–3.3 mya by Blanc and Wolfe [62].

Estimated dates for major lineage divergences implied that 13 taxonomically widespread blocks were of Cretaceous antiquity at least (Table 2). These included the [06-08-10-11] CMA, block 12 (the second I-box in rosids), and blocks 17 (Box II), 23 (CAAT-box), 29 (LRE) and 30 (TATA-box). Another, block 20, remains poorly characterized in functional terms, but does bind protein (Figure 4), and was further noted in genes from the Amaranthaceae (*Spinacia oleracea rbcS-1 *[EMBL X73236, -191 bp]), and Malvaceae (*Gossypium hirsutum rbcS *[EMBL X54091, -186 bp]). Other Cretaceous blocks were three rosid blocks (02, 24 and 35) discovered in the present study. The remaining blocks were found only in single families but could be dated by clade divergence or gene duplication events to 18–54 mya (Table 2).

The occurrence of particular phylogenetic footprints at different levels in the taxonomic hierarchy (Table 2) indicated that the 5'-NCS might be amenable to phylogenetic analysis. Opinions differ, however, about phylogenetic analysis of NCS, particularly at higher taxonomic levels. NCS are seen as problematic for alignment and phylogenetic analysis because of their structural constraints, non-randomness of evolution, and mutational changes such as slipped-strand mispairing, stem-loop secondary structure excision/repair, minute inversions, and intramolecular recombination [9]. In practice, however, Bremer et al. [10] found chloroplast NCS to be of similar utility to coding sequences for asterid phylogenetics.

**Figure 7:**
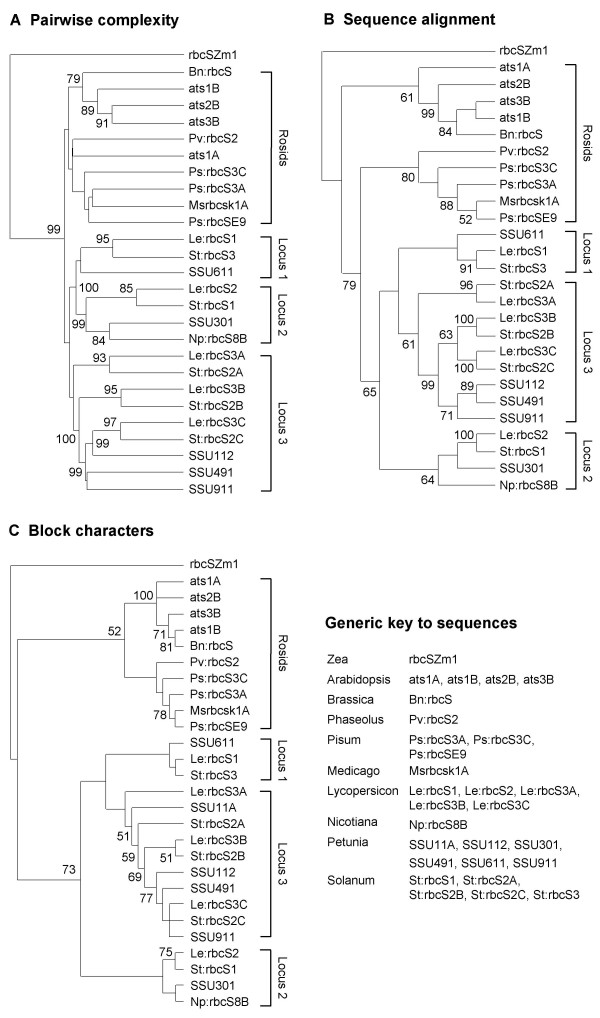
**Dendrograms of *rbcS *5'-NCS relations constructed by 3 methods**. Groupings highlighted for rosid (brassica and legume) genes, and the 3 solanaceous loci. Non-unique gene symbols prefixed with binomial species initials. (A) Hierarchical cluster analysis, with each sequence defined as vector of *C*_*LZ *_values from pairwise decomposition by each of the others. Numerals indicate nodes with multiscale bootstrap resampling values ≥ 50% obtained by PVCLUST. (B) Parsimony analysis by PAUP* of DIALIGN alignments. 50% majority-rule consensus of 234 most-parsimonious trees shown with bootstrap values ≥ 50%. (C) Parsimony analysis by PAUP* of sequences defined by block characters. 50% majority-rule consensus of 882 most-parsimonious trees shown with bootstrap values ≥ 50%.

In view of the technical uncertainties and limited precedents for exploring evolutionary relations between 5'-NCS [9,10], we compared several distinct methodologies. First, given the role of *C*_*LZ *_analysis in our phylogenetic footprinting, comparison of the 5'-NCS based on this methodology was pertinent. A set of *N *sequences can be described in terms of their pairwise complexities, in the form of *N *vectors each containing *N *components. The (*i*,*j*) component is the pairwise *C*_*LZ *_with respect to direct repeats between sequences *i *and *j*. To some extent, pairwise *C*_*LZ *_measures an evolutionary distance between sequences by the number of steps required to produce sequence *j *from sequence *i *using it as a source of building blocks. Hierarchical cluster analysis of 5'-NCS in this format produced the dendrogram in Figure 7A. (As *C*_*LZ *_depends on sequence length, 5'-NCS shorter than the maximum length of 400 bp had the potential to yield anomalous results. Short sequences were therefore analyzed only if overall topology was robust to their inclusion; only *Petunia SSU11A *was omitted in consequence.)

Secondly, more conventional analyses based on DNA parsimony or distance were applied to 5'-NCS aligned using DIALIGN, or CLUSTALW with the gap penalties found to be most effective in phylogenetic footprinting (Figure 1). (ALIGN-M was not usable as it does not produce complete alignments where sequence tracts are too divergent.) Figure 7B shows a consensus of most-parsimonious trees of DIALIGN-aligned 5'-NCS. (The short *Petunia SSU11A *sequence was also omitted from this tree for reasons discussed for Figure 7A.)

Our third method (Figure 7C) was a cladistic analysis of character-states defined as presence or absence of conserved blocks. All blocks in Figures 2, 3 were included: of these 96.9% had ≥ 50% identity with the definitions in Table 1. The remainder averaged 45.6% identity, and all but one had been found by three phylogenetic footprinting methods. Close inspections of aligned locations scored as absences were often suggestive of degenerate residues of blocks.

Several points of congruence between the dendrograms produced by these diverse analyses were identifiable, though bootstrap support for nodes was often moderate or weak (Figure 7). Themes included the clustering of the 5'-NCS by gene loci rather than by species. Thus, 5'-NCS of the *Arabidopsis atsB *tandem locus showed more affinity with the *Brassica *sequence than with *Arabidopsis ats1A*. This accorded with the conclusion of Bowers et al. [59] that the ancestral α duplication event occurred prior to the *Brassica*-*Arabidopsis *split, because 49–64% of relevant *Brassica *genes were more similar to one *Arabidopsis *gene than was the *Arabidopsis *duplicate.

**Figure 8:**
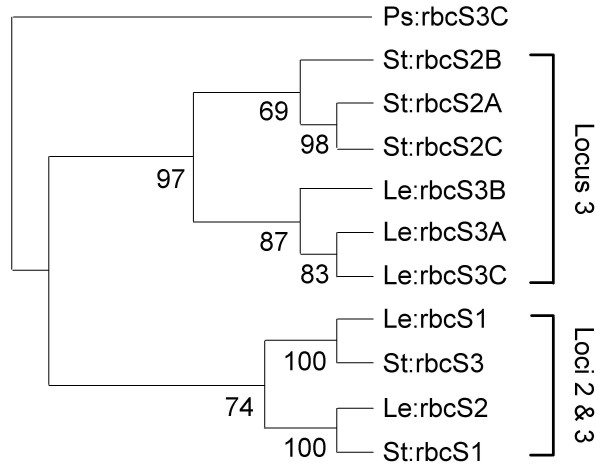
**Parsimony analysis of solanaceous *rbcS *coding sequences aligned by CLUSTALW**. 50% majority-rule consensus of 182 most-parsimonious trees from branch-and-bound search shown with pea *rbcS-3C *as outgroup. Numerals indicate bootstrap values of nodes. Gene labels as Figure 7.

Another theme was the segregation of the solanaceous 5'-NCS as the three loci deduced by Dean et al. [25] (Figure 7). Pairings of tomato and potato orthologues received particularly strong bootstrap support, consistent with a recent speciation [62]. In contrast, the coding sequences of tomato and potato locus3 instead segregated by species (Figure 8). Similar discrepancies between noncoding- and coding-sequence trees in several organisms have been attributed to gene conversion processes that have a greater effect on coding sequences [63]. Also consistent with gene conversion in the locus3 coding sequences were very low intralocus *Ks *values that would imply tandem duplication near the tomato-potato speciation time (Figure 6), which would be hard to reconcile with the more ancient relationships of their 5'-NCS to *Petunia *orthologues (Figure 7). Gene conversion in the *Petunia *locus 3 genes themselves was suggested by Dean et al. [64].

The locus 3 5'-NCS presented a consistent picture in that the tomato and potato *A *genes were resolved as basal members of a monophyletic group (Figure 7). In fact, tomato *rbcS-3A *was the only gene retaining the ancestral [06-08-10-11] CMA in the analyzed region (Figure 3). The most likely counterpart among the *Petunia *5'-NCS analyzed was *SSU11A *(Figure 7C). *Petunia SSU112*, *SSU491 *and *SSU911 *grouped with the more derived 5'-NCS of the tomato and potato *B *and *C *genes.

The remaining solanaceous 5'-NCS grouped into loci 1 and 2 (Figure 7). In evolutionary trees based on CLUSTALW rather than DIALIGN alignments, the *Petunia *locus 1 gene *SSU611 *formed the outgroup to locus 2, while the tomato and potato locus 1 genes grouped with the *A *genes of locus 3 (not shown). The DIALIGN trees were preferred as they were supported by the alternative dendrograms, and because we rated the alignments from this algorithm most highly (Figure 1). Moreover, CLUSTALW alignments of transit peptides supported affinity of *SSU611 *with the other solanaceous locus 1 genes (not shown).

The basal solanaceous locus could not be confidently identified, as the basal position of locus 2 in two of the dendrograms in Figure 7 had only moderate bootstrap support. Clear guidance was not forthcoming from the coding sequence *Ks *values (Figure 6), or from parsimony analyses (Figure 8), in which outgroup choice influenced topology with respect to these two loci.

## Discussion

Conserved blocks revealed by phylogenetic footprinting in dicot *rbcS *5'-NCS formed an evolutionary hierarchy, from those common to plant families that diverged in the Cretaceous, to family-specific blocks with minimum estimated ages of only about 20 million years. Similar heterogeneity in longevity and clade-specificity of promoter motifs has been found in other organisms of ancient divergence. Among homologous human and rodent TF binding sites, for example, Dermitzakis and Clark [65] found 33 with shared functions, while 14 were human-specific and 17 rodent-specific.

The most ancient conserved blocks we found included those recognized earliest in *rbcS *research on the basis of functional importance (I-boxes, G-box, Box II, CAAT-box, TATA-box) [25,27,32], though several relatively unknown ones also fell in this category. Furthermore, we were able to extend CMAs postulated in previous studies [28]. Younger blocks were generally of less widely recognized function, and presumably had acquired roles in the more recent clades in which they had evolved. Simulations by Stone and Wray [66] of the acquisition by point mutation of novel TF binding sites, and their subsequent fixation within populations, indicated the evolution of new sites must be virtually inevitable over millions of years. In a theoretical population of 10^6 ^*Arabidopsis *plants with two generations per year, the fixation time for two 6-bp binding sites in a 200-bp region was only 270,000 years.

Evolutionary information in the 5'-NCS was sufficient for several formal computational methods to produce dendrograms in accordance with the existing classification of solanaceous *rbcS *loci based descriptively on sequence similarities, intron features and linkage relations [25,27,67]. The solanaceous locus 2 genes are distinguished as the only land plant *rbcS *genes with introns at three positions, while locus 3 is a distinctive tandem array of three 2-intron *rbcS *genes in tomato and potato, and probably six in *Petunia*. Gene duplications appear to have provided additional impetus in functional evolution of *rbcS *genes. For instance, the strongly expressed locus 3 tomato genes *rbcS-3B *and *rbcS-3C *[68] represented the most derived members of tandem arrays according to our dendrograms. It has been suggested that gene duplicates are conserved and subfunctionalized by regulatory mutations, because each duplicate must survive to complement lost expression for essential subfunctions in the other [69]. Duplicate gene preservation by such a process could be < 4 million years for a gene with ≥ 5 regulatory elements and a mutation rate of 10^-7 ^per year [69]. Such a rapid preservation of duplicates may need to be invoked for locus 3, because of coincident estimates (18–23 mya) for the major ancestral genome duplication event [62] and for divergence of the *Petunia *clade [51]. In our dendrograms, segregation of *Petunia SSU112*, *SSU491 *and *SSU911 *with the more derived tomato and potato genes of locus 3 indicated that tandem duplications at locus 3 had occurred prior to the *Petunia *divergence, and had undergone relatively little subsequent sequence evolution.

Point mutations do not appear to have been the only evolutionary processes governing protein interactions in the *rbcS *5'-NCS. Mechanisms such as slipped-strand mispairing [9] probably generated the relatively extensive and variable low-complexity tracts that coincided with known DNase footprints in the locus 3 tomato genes. Another example of the gross mutational processes that can occur in 5'-NCS was the *Stowaway-Le2 *transposable element in the tomato *rbcS-1 *sequence (Figure 3). The absence of this transposable element from the potato sequence [57] implies a recent insertion event in tomato.

A primary factor that facilitated our study was a suite of phylogenetic footprinting tools that complemented and cross-validated each other. The least known member of our toolkit was probably *C*_*LZ *_analysis, whose use deserves to increase with its availability as an internet tool [55]. Its intuitive process of sequence decomposition by repeated fragments proved useful not only for identification of conserved motifs, but also for highlighting low-complexity regions such as AT-rich tracts, and as a similarity measure for global sequence comparisons and hence dendogram construction. Otu and Sayood [70] formally examined *C*_*LZ *_as a new sequence distance measure for phylogenetic tree construction, and demonstrated that its lack of dependence on alignments or evolutionary models was particularly suited for sequences subject to segment-based modifications, including whole mitochondrial genomes of eutherian mammals. Promising alternative alignment-independent methods of sequence comparison have also been proposed using the general information theoretical concept of Kolmogorov complexity [71,72], of which *C*_*LZ *_is one explicitly computable implementation.

The dendrograms we produced using *C*_*LZ*_, and those obtained by parsimony analysis of DIALIGN alignments or block characters, were of sufficient consistency to confirm the presence of evolutionary information in plant 5'-NCS. The dataset was not designed to investigate taxonomic phylogenies, as it included several multigene families. Moreover, we would not claim that the dendrograms rival in quality those produced using coding sequences, as bootstrap support for nodes was often moderate or weak, and there were points of variance between the dendrograms. Further investigation is needed to establish the extent to which NCS might contribute to molecular phylogenetics. We do, however, conclude that current computational methods provide the potential for analysis of the evolution of gene expression in terms of promoter structure.

## Conclusion

Comprehensive phylogenetic footprinting of dicot 5'-NCS revealed conserved modular arrays of recurrent sequence blocks. Transcriptional functionality was confirmed as an evolutionary basis for this conservation by coincidence of recurrent blocks with *cis*-elements and protein-binding sites. Evolutionary hierarchies were discernible within the assemblage of blocks, such that taxonomically widespread, and hence ancient, blocks could be distinguished from taxon-specific, more recent, ones.

## Methods

### Database information

Noncoding sequences (NCS) up to 400 bp including and immediately 5' to the ATG codon were obtained for the following genes [accession numbers, bp analyzed]: *Arabidopsis thaliana ats1A *[EMBL:X13611, 400], *ats1B *[EMBL:X14564, 400], *ats2B *[EMBL:X14564, 400], *ats3B *[EMBL:X14564, 400]; *Brassica napus rbcS *[EMBL:X61097, 400]; *Phaseolus vulgaris rbcS-2 *[EMBL:AF028707, 400]; *Pisum sativum rbcS-E9 *[EMBL:X00806, 400], *rbcS-3A *[EMBL:M21356, 400], *rbcS-3C *[EMBL:X04334, 331]; *Medicago sativa rbcSK-1A *[EMBL:X96847, 400]; *Lycopersicon esculentum rbcS-1 *[EMBL:X05982, 338], *rbcS-2 *[EMBL:X05983, 400], *rbcS-3A *[EMBL:X05984, 380], *rbcS-3B *[EMBL:X05985, 283], *rbcS-3C *[EMBL:X05986, 300]; *Petunia × hybrida SSU112 *[EMBL:X12990, 351], *SSU11A *[EMBL:X03821, 281], *SSU301 *[EMBL:X12986, 400], *SSU491 *[EMBL:X12988, 400], *SSU611 *[EMBL:X12987, 400], *SSU911 *[EMBL:X12989, 400]; *Nicotiana plumbaginifolia rbcS-8B *[EMBL:X13711, 400]; *Solanum tuberosum rbcS-1 *[EMBL:X69759, 400], *rbcS-2A *[EMBL:X69760, 400], *rbcS-2B *[EMBL:X69761, 400], *rbcS-2C *[EMBL:X69762, 400], *rbcS-3 *[EMBL:X69763, 382]; *Zea mays rbcSZm1 *[EMBL:S42508, 400]. Coding sequences were from the same accessions, except *P. sativum rbcS-3A *[EMBL:X04333] and *Zea mays ZmrbcS *[EMBL:Y00322].

Duplicated ancestral chromosome segments encompassing the Arabidopsis *ats *genes were identified (as block 0105451100840) in the PARALOGONS IN *ARABIDOPSIS THALIANA *database [58,73]. Potential *cis*-elements in the 5'-NCS were identified using the PLACE [74,75] database.

### Sequence analysis

Recurrent sequence blocks were identified in *rbcS *5'-NCS by Lempel-Ziv complexity (*C*_*LZ*_) decomposition. Lempel and Ziv [76] suggested measurement of sequence complexity by the number of steps required for the iterative generation (recovery) of a given sequence *S *from scratch, using two possible 'recovery' operations per iteration: either copy a fragment that has already been encountered in the recovered part of the sequence; or add (generate) a new symbol not encountered before. This iterative process, called a *decomposition*, represents a sequence *S *as a concatenation of *m *consecutive fragments, *H*(*S*) = *S *[1:*i*_1_]*S *[*i*_1_+1: *i*_2_]...*S *[*i*_*m*-1_+1: *i*_*m *_= *N*], where S [*i*_*k*-1_+1: *i*_*k*_] is a fragment copied or generated at *k*-th step, *N *is the length of the sequence and *m *= *m*_*H*_(*S*) is the number of steps in decomposition process. Among all possible decompositions the one with the minimum number of steps defines sequence complexity, i.e. CLZ(S)=min⁡H{mh(S)}
 MathType@MTEF@5@5@+=feaafiart1ev1aaatCvAUfKttLearuWrP9MDH5MBPbIqV92AaeXatLxBI9gBaebbnrfifHhDYfgasaacH8akY=wiFfYdH8Gipec8Eeeu0xXdbba9frFj0=OqFfea0dXdd9vqai=hGuQ8kuc9pgc9s8qqaq=dirpe0xb9q8qiLsFr0=vr0=vr0dc8meaabaqaciaacaGaaeqabaqabeGadaaakeaacqWGdbWqdaWgaaWcbaGaemitaWKaemOwaOfabeaakiabcIcaOiabdofatjabcMcaPiabg2da9maaxababaGagiyBa0MaeiyAaKMaeiOBa4galeaacqWGibasaeqaaOGaei4EaSNaemyBa02aaSbaaSqaaiabdIgaObqabaGccqGGOaakcqWGtbWucqGGPaqkcqGG9bqFaaa@4287@. The minimum is ensured by copying at each step of the decomposition process the longest fragment that has been encountered before. Similarly, one can define a pair-wise complexity of sequences *S *and *Q*, *C*(*S*|*Q*), as the number of steps needed to recover *Q *from *S *(or *S *from *Q*). In this case, each fragment in the decomposition of *Q *is the longest one whose copy occurs anywhere in sequence *S*. Gusev et al. [15] proposed a linear algorithm for sequence decomposition and computation of *C*_*LZ *_with respect to various types of repeat (including direct and inverted repeats or any combination of them). Its implementation is available online at LZCOMPOSER [55,77].

The full algorithm used in this study follows. **Step 1**. For *N *5'-NCS denoted as *S*_1_,..., *S*_*N *_(with corresponding lengths |*S*_1_|,..., |*S*_*N*_|), a new, concatenated sequence *ŋ *= *S*_1_#...# *S*_*N *_of length *L *= Σ |*S*_*i*_|, *i *= 1,..., *N *was defined. (The arbitrary symbol # separated the concatenated sequences.) **Step 2**. A Lempel-Ziv decomposition of *ŋ *into *m *consecutive fragments, *ŋ *[1:*i*_1_]*ŋ *[*i*_1_+1:*i*_2_] ... *ŋ *[*i*_*m*-1_: *L*], was computed, such that *ŋ *[*i*_*k*-1_+1:*i*_*k*_] was the longest fragment downstream of position *i*_*k-1 *_for which a direct repeat occurred starting from position *j*(*k*) somewhere upstream of position *i*_*k*-1_+1, and *ŋ *[*i*_*k-1*_+1:*i*_*k*_] did not contain #. Pointers *j*(*k*) were expressed as pairs (sequence number, position within the sequence). **Step 3**. Fragments ≥ 8 bp that were common for at least two sequences were included in a vocabulary of 'blocks'. Only exact matches were considered in the decomposition process. However, when two or more consecutive fragments of a decomposition were identical to the respective substrings in another sequence, and when these fragments were separated by a similar number of nucleotides (± 1) then they were merged into a single block. All remaining sequences from the given dataset were scanned for the occurrence of these blocks. For each block, the origin in the decomposition and the entire track of occurrences in different sequences were traced, ensuring that the fragments found were independent of the sequence order in *ŋ*. **Step 4**. Fragments defined as the same block were aligned, including an extra 10 bp either side to check for possible block extension, and their consensus sequence was defined allowing for a given number of mismatches (initially two). **Step 5**. All sequences were then scanned for each block defined by its consensus. No steps involved *a priori *knowledge of *cis*-elements. A similar algorithm was used to search for inverted repeats [16], but we found too few of these for detailed analysis. The decomposition process is available online at LZCOMPOSER [55,77].

Matrices (*N *× *N*) of pairwise *C*_*LZ *_values for *N *sequences were produced on LZCOMPOSER (using the symmetrized matrix output with diagonals adjusted to 0). Sliding-window profiles of local *C*_*LZ *_along single sequences were also generated on LZCOMPOSER.

Overrepresented motifs in the 5'-NCS were also sought using MOTIF SAMPLER v3.1 [21]. The 5'-NCS were analyzed in 19 combinations, with program options in the following ranges: search (s), single stranded; prior probability of motif (*p*), 0.3–0.8; length of motif (*w*), 9–25 bp; number of different motifs (*n*), 3–20; number of instances of each motif per sequence (*M*), 1, 2 or undefined; allowed overlap (*x*), 1–9 bp; program repeat runs (*r*), 0–99. Background models of order 0–3 were used in the analysis.

Multiple alignments of 5'-NCS were performed with three algorithms: CLUSTALW v1.83 [78]; DIALIGN 2 [22] in the QALIGN v1.10T software of Sammeth et al. [79]; and ALIGN-M v2.3 [24]. Unless stated, gap penalties in both CLUSTALW and the S2P step of ALIGN-M were: opening 8.0; extension0.5. In the search process for conserved blocks, a total of 26 different sequence combinations were aligned with DIALIGN and/or ALIGN-M, and the blocks from *C*_*LZ *_analysis and MOTIF SAMPLER were mapped in the alignments.

Levels of synonymous substitutions (*Ks*) were obtained by multiple alignment of all the tomato and potato *rbcS *coding sequences by CLUSTALW (default gap penalties), followed by estimation of the matrix of pairwise *Ks *values by the method of Li [80] implemented in the R package SEQINR [81].

### Dendrograms

Three strategies were used to produce dendrograms of the 27 dicot sequences, with the 5'-NCS of *Zea rbcSZm1 *included as outgroup. (1) Hierarchical cluster analysis was performed on matrices of pairwise *C*_*LZ *_values (see above), using Euclidean distance to measure similarity of the different rows. Dendrograms were produced by the unweighted pair group method with arithmetic mean (UPGMA), using PAST v1.34 [82] and the R package PVCLUST [81]. Statistical support was assessed using PVCLUST to calculate the approximately unbiased (AU) values of Shimodaira [83] by multiscale bootstrap resampling of 1000 pseudoreplications. (2)Evolutionary trees were produced from multiple sequence alignments created with DIALIGN or CLUSTALW. Trees were obtained, using PAUP* v4.0b10 [84] and PHYLIP v3.64 [85], by DNA parsimony or, by the neighbour-joining, UPGMA or Fitch-Margoliash methods, from DNA distance matrices produced with the Jukes-Cantor substitution model. (3)Cladistic analysis, using PAUP* v4.0b10 and PAST v1.34, was performed on the conserved blocks identified by sequence analyses. A character-state matrix of absence (0) or presence (1) of each block was created. Characters were assigned equal weight and Dollo status (i.e. a block could evolve only once, but could disappear at several points on the tree). The tree-bisection-reconnection heuristic was used to search for the most parsimonious topologies. For methods (2) and (3), the *Zea *sequence *rbcSZm1 *was specified as outgroup, and nodal support was estimated from 100 tenfold-replicated bootstrap pseudoreplicates.

Evolutionary trees of coding sequences were obtained by bootstrapped parsimony analysis in PAUP*v4.0b10 of sequences aligned by CLUSTALW (default gap penalties) or DIALIGN.

## List of abbreviations

*C*_*LZ*_, Lempel-Ziv complexity; CMA, conserved modular array; GBF, G-box binding factor; LRE, light-responsive element; *Ks*, level of synonymous substitutions; mya, million years ago; PPV, Positive Predictive Value; NCS, noncoding sequences; TF, transcription factor; UPGMA, unweighted pair group method with arithmetic mean; UTR, untranslated region.

## Authors' contributions

Bioinformatic and dendrogram analyses were carried out by KW, NC and IS. The study was designed and coordinated by NC, ID and IS. The manuscript was drafted by IS with contributions and approval by all authors.

## Supplementary Material

Additional file 1 Conserved blocks in *rbcS *5'-NCS. Alignments and locations of conserved blocks in all sequences. Click here for file
