# Supplementary material for: Evolutionary hierarchies of conserved blocks in 5'-noncoding sequences of dicot rbcS genes
Source: BMC Evol Biol. 2007 Apr 2;7:51. doi: 10.1186/1471-2148-7-51 (PMC1852302; doi:10.1186/1471-2148-7-51)
Supplement: Additional file 1 — Conserved blocks in rbcS 5'-NCS. Alignments and locations of conserved blocks in all sequences. [file 1471-2148-7-51-S1.pdf]

CONSERVED BLOCKS IN *rbcS* 5′-NCS

SEQUENCES

**Code**	**Species**	**Gene**	**(EMBL Accession, bp)**
PsrbcSE9	*Pisum sativum*	*rbcS-E9*	(X00806, 397)
PsrbcS3A	*Pisum sativum*	*rbcS-3A*	(M21356, 397)
PsrbcS3C	*Pisum sativum*	*rbcS-3C*	(X04334, 328)
PvrbcS2	*Phaseolus vulgaris*	*rbcS-2*	(AF028707, 397)
Msrbcsk1A	*Medicago sativa*	*rbcSK-1A*	(X96847, 397)
BnrbcS	*Brassica napus*	*rbcS*	(X61097, 397)
ats1A	*Arabidopsis thaliana*	*ats1A*	(X13611, 397)
ats1B	*Arabidopsis thaliana*	*ats1B*	(X14564, 397)
ats2B	*Arabidopsis thaliana*	*ats2B*	(X14564, 397)
ats3B	*Arabidopsis thaliana*	*ats3B*	(X14564, 397)
LerbcS1	*Lycopersicon esculentum*	*rbcS-1*	(X05982, 335)
LerbcS2	*Lycopersicon esculentum*	*rbcS-2*	(X05983, 397)
ssu301	*Petunia × hybrida*	*SSU301*	(X12986, 397)
rbcS8B	*Nicotiana plumbaginifolia*	*rbcS-8B*	(X13711, 397)
StrbcS1	*Solanum tuberosum*	*rbcS-1*	(X69759, 397)
LerbcS3A	*Lycopersicon esculentum*	*rbcS-3A*	(X05984, 377)
LerbcS3B	*Lycopersicon esculentum*	*rbcS-3B*	(X05985, 280)
LerbcS3C	*Lycopersicon esculentum*	*rbcS-3C*	(X05986, 297)
ssu112	*Petunia × hybrida*	*SSU112*	(X12990, 348)
ssu11A	*Petunia × hybrida*	*SSU11A*	(X03821, 278)
ssu491	*Petunia × hybrida*	*SSU491*	(X12988, 397)
ssu911	*Petunia × hybrida*	*SSU911*	(X12989, 397)
StrbcS2A	*Solanum tuberosum*	*rbcS-2A*	(X69760, 397)
StrbcS2B	*Solanum tuberosum*	*rbcS-2B*	(X69761, 397)
StrbcS2C	*Solanum tuberosum*	*rbcS-2C*	(X69762, 397)
StrbcS3	*Solanum tuberosum*	*rbcS-3*	(X69763, 379)
ssu611	*Petunia × hybrida*	*SSU611*	(X12987, 397)

CONSERVED BLOCKS (-bp from ATG codon)

BLOCK 01

**Definition**	:	GCGTCTGATTT
BnrbcS	-277	GCGTCAGAATT
ats1B	-269	GCATCTGATTT
ats3B	-340	GCGTCTGATTT

BLOCK 02

**Definition**	:	AAGGAGCCAAAAGC
BnrbcS	-250	AAGGAGCCAAAAGC
ats1A	-357	AAGG-GCCAAGTCC
ats1B	-242	AAGGAGCCAAA-GC
ats2B	-311	AAGGAGCCAAAAGC
ats3B	-313	AAGGAGCCAAAAGC
Msrbcsk1A	-330	AAGGAGCCAAA-GC

BLOCK 03

**Definition**	:	AACCGATCAAGTGGAGA
BnrbcS	-236	ATTGGCTCAAGTTGAGA
ats1A	-345	CACCAGGCAAGTAAAAT
ats1B	-229	AACCGATCAAGTGGAGA
ats2B	-297	AACCGATCAAGTGGAGA
ats3B	-299	AACCGATCAAGTGGAGA

```
BLOCK 04
Definition    :          AAAAATGAAAAACTTGTC
LerbcS3C      -297       AAAAATGAAAAACTCGTC
ssu112        -294       AAAGTGATTAAAATTGTC
ssu491        -337       AAAAAGAATTAACTTGTT
ssu911        -300       AAAAATGAAAAACTTGTC
StrbcS2B      -318       TCAAGTGAAGAAATTGTT
StrbcS2C      -296       AAAAATGAAAAACTTGTC

BLOCK 05
Definition    :          AACCATACACA
BnrbcS        -214       AACCATACACA
ats1A         -321       CACCACTCCAC
ats1B         -206       AACCATACACA
ats2B         -275       ATCCATACACA
ats3B         -277       AACCG--CACA

BLOCK 06
Definition    :          ATCACACATT
PsrbcS3A      -284       ATCACACATT
PsrbcS3C      -234       ATCACACATT
PvrbcS2       -307       ATCACACATT
Msrbcsk1A     -306       ACCATACATG
BnrbcS        -203       TTCATACGTT
ats1A         -309       ATCACACAAT
ats1B         -195       ATCACTCATT
ats2B         -264       TTCACTCCTA
ats3B         -268       TTCACTTCAT
LerbcS2       -246       ATCATACATT
ssu301        -337       ATCACACATT
rbcS8B        -367       ATCATACATT
StrbcS1       -275       ATCACCCATT
LerbcS3A      -346       TTCACTCATT
StrbcS3       -360       ATCACACATT
ssu611        -262       ACCAACCATT

BLOCK 07
Definition    :          ATATCCTCTTCCTACCCCCAT
LerbcS2       -235       ATATCCTCTTCCTACCCCCAT
ssu301        -326       ATATCCACTTCCTAC-TCCAT
rbcS8B        -356       ATATCCTCTTCCTACCCCCAT
StrbcS1       -264       ATATCCTCTTCCTACCCCCAT
ssu611        -251       ATATCCTCTTCCTACCCC-AT

BLOCK 08
Definition    :          GATGAGATAAGA
PsrbcS3A      -260       GAAAAGATAAGA
PsrbcS3C      -213       CACATGAAAAGA
PvrbcS2       -283       GAAGAGATAAGA
BnrbcS        -184       GATAAGATAAGA
ats1          -288       ATAACGATAAGA
ats1B         -175       AGAAAGATAAG-
ats2B         -244       AGAAAGATAAGA
ats3B         -245       GAAAAGATAAGA
LerbcS2       -210       GATGAGATAAGA
ssu301        -302       GATGAGATAAGA
rbcS8B        -331       GATGAGATAAGA
StrbcS1       -239       GATGAGATAAGA
LerbcS3A      -321       GATGAGATAAGA
StrbcS3       -341       GATGATATAAGA
ssu611        -227       GATGAGATAAGA
```

```
BLOCK 09
Definition     :            TTTGAGATAAGGA
LerbcS3B      -163          TTTAAGATAAGGA
LerbcS3C      -192          TTTGAGATAAGGA
ssu112        -220          TTTGAGATAAGGA
ssu491        -249          TTTGCGATAAGGA
ssu911        -227          GTTAAGATAAGGA
StrbcS2B      -174          TTTAAGATAAGGA
StrbcS2C      -192          TTTGAGATAAGGA
```

```
BLOCK 10
Definition     :            ACACGTGGCA
PsrbcSE9      -248          ACACGTGGCA
PsrbcS3A      -233          CCACATGGCA
PsrbcS3C      -189          CCACGTGGCA
PvrbcS2       -258          CCACGTGTCA
Msrbcsk1A     -260          ACACGTGGCA
ats1A         -260          CCACGTGGCA
ats2B         -217          CCACGTGATC
ats3B         -218          CCACGTGGCC
LerbcS2       -184          ACACGTGTCA
ssu301        -277          CCACGTGGCA
rbcS8B        -304          ACACGTGGCA
StrbcS1       -213          ACACGTGTCA
LerbcS3A      -295          ACACGTGGCA
ssu11A        -255          CCACGTGGCA
StrbcS3       -313          ACACGTGGCA
ssu611        -201          ACACGTGTCA
```

```
BLOCK 11
Definition     :            TCCTATTGGTGGCT
PsrbcSE9      -232          TCCTATTGGTGGCT
PsrbcS3A      -220          TCCTATTGGTGGCT
PsrbcS3C      -176          TCATAGTGATGCAT
PvrbcS2       -246          TCCACATGGTACCT
Msrbcsk1A     -247          TTATATTGGTGGCT
ats1A         -247          TTCCAGCGGTTCAA
ats2B         -204          TCCTAGTGGTCCAA
ats3B         -205          TCCTAGTGGTGCGT
LerbcS2       -171          CTATTGTGGTGACT
ssu301        -264          CCATTGTGGTGACA
rbcS8B        -291          CCATTGTGGTGACT
StrbcS1       -200          CTATTGCGGTGACT
LerbcS3A      -281          TTCTTGTGGCTTAA
ssu611        -188          CCATTCTGGTTAGT
```

```
BLOCK 12
Definition     :            GATAAGGCT
PsrbcSE9      -215          GATAAGGTT
PsrbcS3A      -203          GATAAGGCT
PsrbcS3C      -160          TGTATGGCT
PvrbcS2       -229          GATAAGGCT
Msrbcsk1A     -230          GATAAGGCT
ats1A         -230          GATAAGGGT
ats1B         -164          -ATAAGGGT
ats2B         -187          GATAAGGGT
ats3B         -188          GATAAGGGT
```

```
BLOCK 13
Definition     :          TCAACACCTTTCCTT
BnrbcS        -162        TCTACACCTTTCTTT
ats1A         -220        TCAACACCTCTCCTT
ats1B         -155        TCAACACCTTTCCTT
ats2B         -177        TCAACACCTTTCCTT
ats3B         -178        TCAACACCTTTCCTT

BLOCK 14
Definition     :          GGCACTTAGCTCCAATT
LerbcS1       -254        GGCACTTAGCTCCAATT
StrbcS3       -255        GACACTTAGCTCCAATT
ssu611        -160        GGCTCATAGCTAAAAAT

BLOCK 15
Definition     :          TTTCCAACC
LerbcS2       -149        ATTCCAACC
ssu301        -219        TTTCCAACC
rbcS8B        -245        TTTCCAACC
StrbcS1       -154        TTTCCAACC

BLOCK 16
Definition     :          AGGGGTTAAA
LerbcS3C      -173        AGGGGTTAAA
ssu112        -193        AGGGGTTAAA
ssu491        -222        AGGGGTTAAA
ssu911        -200        AGGGGTTAAA
StrbcS2C      -173        AGGGGTTAAA

BLOCK 17
Definition     :          ATCTTGTGTGGTTAAT
BnrbcS        -148        TAATCCTGTGGCAGTT
ats1B         -141        TAATCATGTGGTAGTG
ats2B         -163        TAATCCTGTGGCAATT
ats3B         -164        TAATCCTGTGGCAGTA
PsrbcSE9      -188        ATCTTGTGTGGTTAAT
PsrbcS3A      -177        ATCTTGTGTGGTTAAT
PsrbcS3C      -131        AACTCGTGTGGTTAAT
PvrbcS2       -202        GACTCGTGTGGCCAGT
Msrbcsk1A     -205        TTCCTGTGTGGTTGAT
LerbcS2       -139        TTCATATGTAGATATT
ssu301        -209        TTCATGTGTGGATATT
rbcS8B        -235        TTCATGTGTGGATATT
StrbcS1       -144        TTCATATGTAGATATT
LerbcS3B      -144        AATTTCAGAGGCTATT
StrbcS2B      -155        AATTTCAGAGGCTATT
LerbcS3C      -165        AAATTCAGTGGCCATT
StrbcS2C      -165        AAATTCAGTGGCCATT
ssu112        -184        AATTCATGTGGCCACT
ssu491        -213        AATTCATGTGGCCACC
ssu911        -192        AATTCATGTGGGCCTT
LerbcS1       -213        CTTTCATGTGGCCATT
ssu611        -133        CTTTCATGTGGCCATT
StrbcS3       -232        CTTTCATGTGGCCATT
StrbcS2A      -196        GTGTATCGTGGTTATT
```

BLOCK 18

**Definition**	:	AACGACGTTATCATGAAT
BnrbcS	-132	AACGACGATATCATGAAA
ats1A	-127	CACTTGAATCTCATGTAT
ats1B	-125	AACGA-GTTATCATGAAT
ats2B	-147	AACGACGTTATCATGAAT
ats3B	-148	AACGACGTTATCATGAAT

BLOCK 19

**Definition**	:	GCAAAGTTT
PsrbcSE9	-166	GCAAAGTTT
PsrbcS3A	-155	GCAAACTTT
Msrbcsk1A	-183	GCAAAGTTT

BLOCK 20

**Definition**		TGTAATGTCA
PvrbcS2	-182	TGTAATGTCA
LerbcS1	-209	TGTAATATAT
LerbcS2	-111	AATGTTATCA
ssu301	-188	TGTAATATCA
rbcS8B	-209	TAATGTATCA
StrbcS1	-116	AATGTTATCA
LerbcS3A	-160	TGAAATGTGA
LerbcS3B	-123	TGAAATGTCA
LerbcS3C	-144	TGTAATGCCA
ssu112	-163	TGTAATGTCA
ssu11A	-194	AGTAATGTCA
ssu491	-192	AGTAATGTCA
ssu911	-171	TAGCAATTCA
StrbcS2A	-174	TTAAATGTCA
StrbcS2B	-134	TGAAATGTCA
StrbcS2C	-144	TGTAATGCCA
StrbcS3	-210	CGTAATATCA
ssu611	-112	TGTAAAATCA

BLOCK 21

**Definition**		ATCATTTTCAC
PsrbcSE9	-157	ATCATTTTCAC
PsrbcS3A	-146	ATCATTTTCAC
PsrbcS3C	-100	ATCATTTTCAC
PvrbcS2	-173	ATCACTTATAG
Msrbcsk1A	-174	ATCATATTCAC

BLOCK 22

**Definition**	:	CCACATAA
LerbcS1	-196	CAACCAAA
LerbcS2	-97	CCACATAA
ssu301	-174	CCACATAA
rbcS8B	-195	CCACATAA
StrbcS1	-102	CCACATAA
LerbcS3B	-109	CCACATAA
LerbcS3C	-130	CCACAAAA
ssu112	-149	CCACATAA
ssu11A	-181	ACACATAA
ssu491	-178	CCACATTA
ssu911	-141	CCACATAA
StrbcS2B	-120	CCACATAA
StrbcS2C	-130	CCACATAA
StrbcS3	-196	CCACATAA
ssu611	-98	CCACATAA

BLOCK 23

**Definition**	:	TCCAATGGTTA
PvrbcS2	-160	TCCGACGGTTG
LerbcS1	-188	TCCAATGGTCG
ssu301	-166	TCCAATGGTTA
rbcS8B	-187	TCCAATGGTTA
LerbcS3A	-138	TCCAATGGTTA
LerbcS3B	-101	TCCAATGGTTA
LerbcS3C	-122	TCCAATGGTTA
ssu112	-141	TCCAATGGTTA
ssu11A	-173	TCCAATGGTTA
ssu491	-170	TCCAAATGGTT
ssu911	-133	TCCAAATGTTA
StrbcS2A	-152	TCCAATGGTTA
StrbcS2B	-112	TCCAATGGTTA
StrbcS2C	-122	TCCAATGGTTA
StrbcS3	-188	TCCAATGGTCA
ssu611	-90	TGCAAAGTTTA

BLOCK 24

**Definition**	:	ACCCTTTGATCATTA
BnrbcS	-109	ATCCTTCGATCATTA
ats1A	-104	ACCCTCCGATCACTC
ats1B	-103	ACCCTTTGATCATTA
ats2B	-124	CCCCTTTGATCATTA
ats3B	-125	ACCCTTTGATCATGA

BLOCK 25

**Definition**	:	TCTAAGATGAGGTTTGCT
LerbcS1	-170	TCTAAGATGAGGCTTCTT
ssu301	-148	TCCAAGA-GAGGTTAGTT
rbcS8B	-169	TCCAAGATGAGGGGGTTG
LerbcS3A	-126	----AAATG-GGTTGGTT
LerbcS3B	-83	TCTTAGATGAGGTTATTG
LerbcS3C	-104	TGTAAGATGAGGTTTGCT
ssu112	-123	TCTAATTAGATGAGGTTT
ssu11A	-155	TCCAAGATGAGGCTTGTT
ssu491	-147	TTTAAGATGAGGTTTCCT
ssu911	-115	TCTAAGATGAGGTTTGCT
StrbcS2A	-134	ACTAA-ATGAGGGTTGGT
StrbcS2B	-94	TCTTAGATGAGGTTATTG
StrbcS2C	-104	TCTAAGATGAGGTTTGCT
StrbcS3	-170	TCAAAGATGAGGTTTCCT

BLOCK 26

**Definition**	:	TACCACAATTT
PsrbcSE9	-113	TACCACAATTT
PsrbcS3A	-101	TACCACAATAT
Msrbcsk1A	-136	TACCACAATTT

BLOCK 27

**Definition**	:	ACCATAATATTGGAA
PsrbcSE9	-98	ACCATAATATTGGAA
PsrbcS3A	-86	ACCATAATATTGGAA
Msrbcsk1A	-120	ACCATAATATTGGAA

BLOCK 28

**Definition**	:	TTGTGTCCGTTAGATG
LerbcS1	-148	TTCTATCCGTTAGATT
ssu301	-128	TTTTGTCCGTTAGATA
rbcS8B	-146	TTTTGTCCGTTAGATA
LerbcS3A	-110	TTGTGTCCGTTAGATG
LerbcS3C	-80	TTTTGTCCGTTAGATA
ssu112	-99	TTGTGTCCGTCAGATG
ssu11A	-134	TTGTATCCGTTAGATG
ssu491	-125	TTGTGTCCGTTAAATG
ssu911	-93	TTGTGTCCGTTAGATG
StrbcS2A	-113	TTGTGACCGTAAGATG
StrbcS2B	-72	TTGTATCCTTTATGTG
StrbcS2C	-80	TTTTGTCCGTTAGATA
StrbcS3	-148	TTCTATCCGTTAGATT

BLOCK 29

**Definition**	:	CCTTATCAT
LerbcS2	-78	CCTTATCAT
StrbcS1	-84	CCTTATCAT
BnrbcS	-76	GCTTCTCAC
ats1A	-70	TGTTATCAT
ats1B	-70	GGTTCTCAC
ats2B	-91	GGTTCCCAC
ats3B	-91	GGTTCTCAT
LerbcS1	-115	CCTTATCAT
ssu301	-95	CCTTATCAT
rbcS8B	-114	CCTTATCAT
LerbcS3A	-78	CCTTATCAT
LerbcS3B	-53	CCTTATCAT
LerbcS3C	-59	CCTTATCAC
ssu112	-66	CTTTATCAT
ssu11A	-102	GCTTAACAT
ssu491	-94	CCTTATCAC
ssu911	-61	CCTTATCAC
StrbcS2A	-81	CCTTATCAT
StrbcS2B	-53	CCTTATCAT
StrbcS2C	-59	CCTTATCAC
StrbcS3	-115	CCTTATCAT
ssu611	-53	CCTTTTCAT

BLOCK 30

**Definition**	:	TATATAAA
PsrbcSE9	-65	TATATAAA
PsrbcS3A	-53	TATATATA
PsrbcS3C	-42	TATATATA
PvrbcS2	-83	TATATAAA
Msrbcsk1A	-87	TATATATA
BnrbcS	-67	TATATATA
ats1A	-61	TATATATA
ats1B	-61	TATATAAA
ats2B	-82	TATATAAA
ats3B	-82	TATATAAA
LerbcS1	-106	TATATAAA
LerbcS2	-64	TATATAAA
ssu301	-86	TATATAAA
rbcS8B	-105	TATATATA
StrbcS1	-70	TATATAAA
LerbcS3A	-69	TATATATA
LerbcS3B	-41	TATATATA
LerbcS3C	-50	TATATATA

ssu112	-57	TATAAATA
ssu11A	-93	TATATATA
ssu491	-85	TATAAATA
ssu911	-52	TATAAATA
StrbcS2A	-72	TATATATA
StrbcS2B	-41	TATATATA
StrbcS2C	-50	TATATATA
StrbcS3	-106	TATATAAA
ssu611	-44	TATATATA

BLOCK 31

**Definition**	:	GAGGGGGA
rbcS8B	-97	GAGTGGTG
LerbcS3A	-61	GAGGGAGA
LerbcS3B	-33	CAAGGGGA
LerbcS3C	-42	CAAGGCGT
ssu112	-49	GAGGGGGA
ssu11A	-85	GAAGGGGG
ssu491	-77	GAAGTGGG
StrbcS2A	-64	GAGGGGGA
StrbcS2B	-33	CAAGGGGA
StrbcS2C	-42	CAGGGGTT

BLOCK 32

**Definition**	:	ATGACAAAACCA
BnrbcS	-58	ATAACCAAAGCA
ats1A	-52	ATGACCAAAGCA
ats1B	-52	ATGACAAAACCA
ats2B	-73	ATGACAAAACCA
ats3B	-73	ATGACAACACCA

BLOCK 33

**Definition**	:	AAGCTTTGCAA
PsrbcSE9	-43	AAGCTTTGCAA
PsrbcS3A	-31	AAGCTTTGCAA
PsrbcS3C	-27	AAGCTTTGCAA
PvrbcS2	-61	AAGCTTGGGCA
Msrbcsk1A	-65	AAGCTTTGCAA

BLOCK 34

**Definition**		GCAATAACCCTCTT
LerbcS1	-82	GTAATAAGCATCTT
StrbcS3	-82	GTAATAAGCATCTT
ssu611	-20	GCAATGACCATCAT
LerbcS3A	-44	GCAATAACCCTCTT
ssu11A	-68	TCAATAACCCTCTT
ssu491	-54	CCATTGACCCTCTT
StrbcS2A	-47	GCAATAACCCTCTT

BLOCK 35

**Definition**		AAGAAGAAGA
PvrbcS2	-18	AAGAAGAAGA
BnrbcS	-20	AAGAAGAAGA
ats1A	-13	GTAAAGAAGA
ats1B	-20	AAGAAGAAGA
ats2B	-29	AAGAAGAAGA
ats3B	-29	AAGAAGAAGA

BLOCK 36

**Definition**		TTTTCAGCA
LerbcS3B	-9	TTGTCAACAATG
StrbcS2B	-9	TTGTCAACAATG
LerbcS3C	-9	ATTTCAGCAATG
StrbcS2C	-9	ATTTCAGAAATG
LerbcS3A	-9	TTTTCAGCAATG
ssu112	-9	ATTTCAGCAATG
ssu11A	-9	TCTTTAGCAATG
ssu491	-9	TTTTCAGAAATG
ssu911	-9	TATTTAGCAATG
StrbcS2A	-9	CACTCAGAAATG
